# RETRACTED: Zhu et al. Synergistic Effect of Bioactive Anticarcinogens from Soybean on Anti-Proliferative Activity in MDA-MB-231 and MCF-7 Human Breast Cancer Cells In Vitro. *Molecules* 2018, *23*, 1557

**DOI:** 10.3390/molecules30214194

**Published:** 2025-10-27

**Authors:** Yingying Zhu, Yang Yao, Zhenxing Shi, Nadia Everaert, Guixing Ren

**Affiliations:** 1 Institute of Crop Science, Chinese Academy of Agricultural Sciences (CAAS), 80 South Xueyuan Road, Haidian, Beijing 100081, China; 467171720@qq.com (Y.Z.); yaoyang@caas.cn (Y.Y.);; 2 Precision Livestock and Nutrition Unit, Gembloux Agro-Bio Tech, TERRA Teaching and Research Centre, University of Liège, Passage des Déportés 2, 5030 Gembloux, Belgium; 3 Laboratory of Biomass and Green Technologies, Gembloux Agro-Bio Tech, University of Liege, Passage des Déportés 2, 5030 Gembloux, Belgium

The Journal retracts the article “Synergistic Effect of Bioactive Anticarcinogens from Soybean on Anti-Proliferative Activity in MDA-MB-231 and MCF-7 Human Breast Cancer Cells In Vitro” [1] cited above.

Following publication, concerns were brought to the attention of the Editorial Office regarding the integrity of Figure 6 presented in this article [1].

Adhering to our standard procedure, an investigation was conducted by the Editorial Office and Editorial Board that identified indications of inappropriate editing and duplication within Figure 6 presented in this article [1]. While the authors cooperated during this process, complete raw material that met the journals requirements (https://www.mdpi.com/journal/molecules/instructions#oriimages) could not be provided for Editorial Board Evaluation. Consequently, the Editorial Board has lost confidence in the reliability of the findings and decided to retract this publication [1], as per MDPI’s retraction policy (https://www.mdpi.com/ethics#_bookmark30).

This retraction was approved by the Editor-in-Chief of the journal *Molecules.*

The authors agreed to this retraction.
